# Incorporating and evaluating an integrated gender-specific medicine curriculum: a survey study in Dutch GP training

**DOI:** 10.1186/1472-6920-9-58

**Published:** 2009-09-08

**Authors:** Patrick W Dielissen, Ben JAM Bottema, Petra Verdonk, Toine LM Lagro-Janssen

**Affiliations:** 1 Department of Primary Care and Community Care, Radboud University, Nijmegen Medical Centre, PO Box 9101, intern code 166, 6500 HB Nijmegen, the Netherlands; 2 Faculty of Health, Medicine and Life Science, Social Medicine, Maastricht University, PO Box 616, 6200 MD Maastricht, the Netherlands; 3 Department of Primary Care and Community Care/ Women's Studies in Medicine, Radboud University, Nijmegen Medical Centre, PO Box 9101, intern code 118, 6500 HB Nijmegen, the Netherlands

## Abstract

**Background:**

We recently set standards for gender-specific medicine training as an integrated part of the GP training curriculum. This paper describes the programme and evaluation of this training.

**Methods:**

The programme is designed for GP registrars throughout the 3-year GP training. The modules emphasize interaction, application, and clinically integrated learning and teaching methods in peer groups. In 2005 - 2008, after completion of each tutorial, GP registrars were asked to fill in a questionnaire on a 5-point Likert scale to assess the programme's methods and content. GP registrars were also asked to identify two learning points related to the programme.

**Results:**

The teaching programme consists of five 3-hour modules that include gender themes related to and frequently seen by GPs such as in doctor-patient communication and cardiovascular disease. GP registrars evaluated the training course positively. The written learning points suggest that GP registrars have increased their awareness of why attention to gender-specific information is relevant.

**Conclusion:**

In summary, gender-specific medicine training has been successfully integrated into an existing GP training curriculum. The modules and teaching methods are transferable to other training institutes for postgraduate training. The evaluation of the teaching programme shows a positive impact on GP registrars' gender awareness.

## Background

Gender-specific medicine (GSM) studies the relationship between gender and health. It is concerned with the promotion of equal opportunity and fair treatment of men and women and aims to redress current gender disparities or gender bias in the healthcare system. Both genders will benefit when GPs deliver healthcare based on education on the role of sex and gender in health and illness. [1]

Various studies have revealed the importance of considering sex and gender issues when providing patient care in general practice. For instance, gender has implications in the presentation of chronic obstructive pulmonary disease (COPD). Women with COPD show higher levels of anxiety and depression and worse symptom-related quality of life than their male counterparts[2,3] A healthcare view focused on men is not unusual in medical decision-making. For example, under-representation of women in studies on cardiovascular disease (CVD) and misinterpretation of women's CVD symptoms results in inadequate diagnoses and suboptimal management in women. [4,5] Furthermore, the physicians' gender as well as patients' gender influence medical communication. Female physicians appear to be stronger in relational communication and conversation facility with their patients which explains partially the higher satisfaction for female physicians. [6] Studies reveal that physicians and medical schools are ill-prepared to recognize these gender-related factors in patient-doctor encounters. [7-9] These findings imply the necessity to train physicians gender issues in GP training.

In the last decade, the field of gender-specific medicine has focussed on training educational professionals, and on reforms aimed to include gender aspects in the curricula of medical schools, and in health research. [10-12] Although many medical educators have called for medical schools to institute training in gender issues, most medical institutions have confined the teaching of gender-specific medicine to optional courses or electives that lack the structure and recognized place to ensure success. [13,14] Innovative ideas to position gender-specific medicine into the medical curriculum often failed to gain long-term commitment from those involved and failed to spread comprehensively throughout their target organizations. [9,15,16]

The influence of gender of physician and patient occurs on all levels of medical encounters and it often comes about unintentionally. Education on knowledge about gender-related processes will likely not be enough to prevent gender disparities in patient care. It is also necessary to address physicians' attitudes and preconceived notions about roles of men and women. The complexity of teaching the subject gender necessitates to go beyond biomedical factors and to include the social context of men and women. That acknowledgement implies that a full integration of a training programme about gender issues may be a necessary condition to getting change and acceptance among GP registrars. One of the methods medical educators can use to ensure future GPs' competency in gender issues is to offer training over time, with reflection on the action, and integrated into clinical practice. [17] Interactive and clinically integrated teaching and learning activities have been shown to be more effective in improving knowledge, skills, attitudes and behaviour in postgraduates than standalone teaching. [18] Although there is a growing awareness of the importance of gender-specific medicine training, to our knowledge no study has documented an integral curriculum on gender-specific medicine in GP training.

Recently, the department of Women's Studies Medicine and the Institute of Postgraduate Training in General Practice at Radboud University Nijmegen Medical Centre launched an initiative for the development of an interactive, long-term training programme in gender-specific medicine. Our goal as teachers is to move GP registrars along a gender sensitivity scale and to (a) enable GP registrars to understand basic concepts of gender, (b) sensitize GP registrars to gender as determinant of health, and (c) ensure that GP registrars attain a reasonable understanding of why gender-specific medicine is relevant. These goals are in line with recent consensus statements which underscore the significance of gender as a key determinant of health and advocate gender equity[19,20]

We developed and evaluated an integrated, interactive training programme to teach GP registrars about gender-specific medicine. In this paper we describe and evaluate this training programme.

## Methods

### Context and objectives

The training programme was developed by four GPs with expertise in and a commitment to gender issues. They synthesized and outlined the training programme in close collaboration with the department of Women's Studies Medicine at Radboud University Medical Centre. The group worked on the specific areas to be covered and educational methods to be used. The experts have been guided by the following questions:

1. Is the topic relevant for and frequently seen by GPs?

2. Does the topic have gender-specific aspects that impact practice?

3. Is there underlying evidence for the gender-specific aspects?

They selected cross-disciplinary topics: cardiovascular disease, depression/anxiety disorders, urinary incontinence, addiction to alcohol or benzodiazepines, domestic and sexual violence. They also included communicative aspects in the training programme. In addition, for each topic they defined a set of objectives, and translated the relevant information and evidence from literature into educational material based on the principles of problem-based learning. Our framework for gender needs assessment is partially based on gender concepts written by Phillips [21]

After the course, GP registrars will be able to:

1. consider the ways in which gender aspects and differences have an impact on health and illness, and an understanding of how gender-related issues may bias the provision of healthcare.

2. consider gender aspects and gender differences in epidemiology, presentation, diagnostic management, and treatment strategies in primary care.

3. understand the impact of doctors' gender in relationships and communication, and model reflective practice around gender issues.

### Learning and teaching methods

Teaching and learning in general practice takes place primarily at work. The key to productive learning in postgraduate training is to focus on the experiences of GP registrars in their practice and to connect theoretical education to these experiences[22] The tutorials are founded on adult learning theory and emphasize interaction, application, reflection, and problem-centred learning. Recent new insights in effective medical education support this strategy. [23] For our training programme it means that we interrelate and clarify gender issues for health conditions in actual contact with patients (e.g., knowledge, judgement, norms). We try to identify and discuss factors that impede change in behaviour toward gender issues (e.g., gender stereotyped perceptions). We promote discussion about approaches to provide appropriate gender sensitive care. In the tutorials we propose that identification and removal of gender-related barriers (e.g. resistance) are important steps toward improving change. Substantial evidence supports interactive and clinically integrated teaching over other teaching methods. [24]

Reflection on gender issues supports GP registrars to understand complex situations by considering them in a larger context, and to identify their particular needs. [25,26] Reflection is a key feature of our training programme. We use reflection to know what registrars do, or neglect to do, with the role of sex and gender in daily practice. Reflection is used in a way of gaining access to perceptions and judgements on gender issues that often escape our awareness.

We work in small-group sessions with 10 to 15 GP registrars who are familiar with each other to facilitate comfort. We used a mix of video-consultations, paper cases, role plays with simulated patients, and reading gender-related articles or narratives. We choose different methods of learning over time as a variety of educational experiences can be more stimulating.

Our GP-supervisors have special skills and specific expertise in teaching gender-specific medicine. They know available resources on gender-specific medicine. Furthermore, they are familiar with resistance and know how to cope with defensive postures of GP registrars when they touch gender issues.

### Structure

The training programme consists of five 3-hours tutorials. An introductory tutorial discusses the intent of the course including the basic concepts of gender, and four specific tutorials address clinical topics in general practice. The sequence used within the tutorials proceeds from an introduction of the topic and an icebreaker exercise, followed by reflection upon experiences and stimulation of self-assessment, to an interactive assignment with a plenary discussion. Typically, the tutorial ends with an overview of the knowledge acquired.

### Content of the curriculum

Tutorial one and two of our training programme are followed in first year (general practice), tutorial three in second year (hospital, psychiatric department, and nursery home), and tutorial four and five in third year (general practice) of GP training. The key features of each tutorial are shortly outlined hereafter and the main factors are presented in table 1.

**Table 1:** The main factors of the gender-specific medicine curriculum in GP training

**Tutorial theme**	**Main objectives**	**Teaching methods**	**Examination acquired knowledge and/or skills**
1. Gender and Socialization	1. be able to understand the concept of gender 2. be able to initiate a gender perspective in medical encounters 3. awareness of the existence of gender socialization and its implications for health issues	-a discourse on the subject (lecture) -group analysis of a video consultation - group reflection on subject with regard to content and process	- questioning by supervisor -identifying learning points
2. Gender and communication	1. understanding of the influence of gender in doctor- patient communication 2. understanding of how gender influences the process of medical-decision making 3. demonstrating gender-sensitive doctor -patient communication	-a discourse on the subject (lecture) -role play with simulation patients(SP) -group reflection on subject with regard to content and process	-questioning by supervisor -assessment and feedback by SP -identifying learning points
3. Gender and psychiatric disorders	1. be able to describe gender differences in depression, anxiety disorders, and substance abuse 2. be able to identify gender differences in social expectations with regard to substance abuse 3. be able to recognize male and female presentation and coping in depression and alcohol abuse	-a discourse on the subject (a lecture) -group reflection on subject with regard to content and process -analysis of case-reports	-questioning by supervisor -identifying learning points
4. Gender and cardiovascular diseases/urinary incontinence	1. be able to understand the gender bias in the care for patients with cardiovascular disease 2. a willingness and ability to minimize the effect of gender bias in cardiovascular disease management 3. be able to describe and recognize the gender differences in presentation and management of urinary incontinence	-pretest to assess gender-specific knowledge -a lecture of gender differences on the subject -group analysis of a video consultation	-pretest and posttest to assess knowledge -questioning by supervisor -identifying learning points
5. Gender and sexual abuse	1. be able to describe the patters and common presentations of sexual violence 2. to increase awareness of sexual violence, potential gender prejudices, and consultation skills 3. be able to demonstrate gender-sensitive consultation skills to promote case-finding of sexual abused patients	-a discourse on the subject (lecture) -role play with SP - group reflection on subject with regard to content and process	-questioning by supervisor -assessment and feedback by SP -identifying learning points

Tutorial one, *gender and socialization*, introduces the concepts of gender and sex. The purpose is to initiate a gender issue perspective into GP registrars' medical encounters. For example, gender differences in life experiences and the influences of family, peers, and media on gender roles. Factors of gender-related attitudes and themes with regard to doctor-patient encounters are discussed to help facilitate a heightened level of gender awareness.

Tutorial two,*gender and communication*, focuses on eliciting the influence of gender on doctor and patient communication and how stereotyped expectations of men and women can affect doctor-patient relationships. Gender differences are addressed that can cause misunderstanding and that can hamper communication between dyads of men and women. An overview of potential gender-related pitfalls in doctor-patient communication is given.

Tutorial three consists of two parts of one and a half hour each: *(a) gender in depression and anxiety disorders, and (b) abuse of alcohol or benzodiazepines*. In this tutorial we address one's own beliefs, norms and values with regard to gender that can influence the provision of care to others. Also we focus and clarify the differences of social expectations for appropriate behaviours of men as compared to women as is the case for alcohol consumption.

Tutorial four deals with *gender differences in cardiovascular disease (CVD) and urinary incontinence (UI)*. Here, we explain the persistent gender differences in cardiovascular disease and the potential biases in the care for patients with CVD such as the stereotypical conceptualisation of CVD as a male disease. GP registrars are taught the importance to reflect on their own and others interpretations, reactions, and conduct in patient care with regard to coronary risk factors in men and women. We address gender differences in patients' beliefs with urinary incontinence for example despite incontinence in men being less severe they experience more distress than women.

Tutorial five, *recognizing and responding to sexual abuse*, addresses sexual violence, a serious and widespread problem for women with a number of social and gender-related barriers that make it hard for GPs to identify such abuse. For example doctor's availability for abused women differs by gender as female doctors tend to restrict their availability due to distress it brought about and male doctors because of time constraints.

### Programme Evaluation

After each tutorial, GP registrars were asked to indicate their level of agreement with initially 5 and later 7 statements to evaluate the course. Each statement was designed to assess the quality of and their opinion on the learning and teaching methods, the perceived relevance for practice, and the usefulness of the applied knowledge. GP registrars' participation was voluntary. We did not assess demographic features with the exception of their sex. We used Likert scales where 1 = totally disagree, and 5 = totally agree. Data were analysed in the SSPS 16.0. Answers were dichotomized so that a response of 1, 2 or 3 suggested a rejection of the program and a response of 4 or 5 implied acceptance of the programme. Significance (*p *< 0.05) was assessed with the use of Chi square test. Similarly, the learning points were evaluated after each tutorial. They were coded and analysed according to the three objectives of the course by the first author (PD).

### Ethical approval

This study fell within the domain of programme evaluation. Consequently ethical approval was not required according to the current regulations at our university.

## Results

In the period February 2005 - September 2008, we collected 442 surveys (response rate 49%). 32,8% of the GP registrars were male (n = 145), 64,7% were female (n = 286). 11 GP registrars did not disclose their sex (2,5%).

The methodology and the supervisors of the GSM programme were well received by the GP registrars. GP registrars' evaluation of the learning methods was in average positive. Overall, more than 80% of the GP registrars positively rated the learning methods used in the programme, the supervisor's role and the approach of the topic. Also, GP registrars appraised gender issues as significant for their learning programme (male 79.5% vs. female 87.2%; X^2 ^= 2.42; ns). Gender-specific information provided in the programme was highly beneficial to their practice (male 82.1% vs. female 89.6%, X^2 ^= 2.72; ns). There were no significant gender differences between the evaluations of the programme but female GP registrars valued the programme consistently higher than male GP registrars.

GP registrars noted 743 learning points on 442 readable evaluation forms. Three main themes were identified in the GP registrars' learning points: *gender as a determinant of health, gender bias in healthcare, and gender in communication and relationships*. Many learning points were about gender differences in epidemiology, presentation, and treatment strategies in primary care (male 39% vs. female 41%). General comments included an increasing awareness of gender issues in general practice or gender-related disease presentation. The learning points dealing with gender bias were about diagnostic management, current lack of gender knowledge and dealing with delicate situations as sexual violence. The theme "understanding of gender bias in healthcare" was almost equal mentioned by male and female GP registrars (male 34% vs. female 31%). Also, both male GP registrars as well as female GP registrars (male 27.1% vs. female 27.3%) described learning points concerning differences between male and female patients and doctors in communication and working patterns. There were remarks about traditional gender roles of doctors and patients in their consultation e.g. in the perception of disease presentation.

Although the evaluation did not confirm negative attitudes about the gender-specific programme on a regular basis, GP registrars did report negative comments on the evaluation forms. For example, some male as well as female GP registrars were of the opinion that the teacher occasionally focussed too much on the backlog of women. Both male and female GP registrars made negative comments on the evaluation forms from time to time that fit into all the types of resistance against gender issues (e.g. simplifications, avoidance, and neglect).

## Discussion

This paper describes the development and pilot-evaluation of a teaching programme in gender-specific medicine for GP training. We present a successful implementation of a mandatory training programme, extended over time, and integrated in the full three-year period of the Dutch GP training. Both male and female GP registrars evaluated the training in gender-specific medicine positively and were satisfied with the education. GP residents considered the education relevant for their learning and daily practice. Based on these outcomes, it demonstrates the feasibility and usefulness of incorporating gender-specific medicine in the curriculum of GP training on a regular basis.

To the best of our knowledge, this is the first programme for on-going teaching and learning of GSM in GP-training. Much of the literature on learning and teaching GSM has been descriptive in nature, providing objectives, goals and theoretical models but not offering mandatory training [27,28] A lack of institutional support and resistance or lack of interest by university departments are problems described in the literature that hinder attempts to introduce a gender perspective in medical education. [29-32] Indeed, our teaching programme is successful partly because it is consistently backed by institutional support. Senior institutional leaders are directly involved in defining goals and objectives, educating and recruiting supervisors, and ensuring appropriate financial support. Furthermore, we have on-going access to educational materials about medical needs of men and women for faculty development. Institutional support, skilled supervisors, and availability of gender-specific information made it possible to embed the GSM teaching programme as a mandatory part of the GP training curriculum - which is undoubtedly beneficial to the teaching results.

Our GSM teaching programme is aimed at all GP registrars and it intends to offer a suitable framework to address gender-specific medicine. Therefore the teaching programme provided a comprehensive overview of medical conditions pertinent to both men and women in primary care. Usually it is *either *women *or *men in teaching gender issues in medical education[33,34] We found that both female and male GP registrars were keen about gender-specific medicine. Female GP registrars evaluated the GSM teaching programme slightly more positively than their male colleagues. It is in line with previous positive results about teaching gender awareness in medical education at Radboud University [35] Nevertheless, a lack of interest in gender issues has also been reported previously from students as well as physician teachers elsewhere. [36,37] Introducing novel and innovative education like gender-specific medicine will undoubtedly involve resistance, neglecting or doubt among GP registrars. We interpreted the negative comments of our GP registrars to the novelty of the field of gender issues in medical education and challenged us continuously to rethink about how we could best teach and engage GP registrars in gender issues. It also stimulated us to employ well trained GP supervisors who are trained in approaches to overcome resistance or barriers of GP registrars.

We feel that the success of our teaching programme is also based on the light the programme sheds on the complex way in which men and women are advantaged and disadvantaged by biological and social factors in health issues and the successful clarification of it by the programme. The programme captured the specific gender-related processes and pathways that leads to health outcomes and emphasized strongly on gender socialization. It went beyond the biomedical framework and discussed gender-related life-experiences concerning health and disease. [38,39] We expanded the historical concept that women's health only relates to reproductive hormones and organs to issues of gender-related medicine of which both men and women can benefit. We wanted to make the case that gender issues in health and in everyday choices and understanding these issues requires integrating social, psychological, and biological perspectives in education. This educational view was in line with evidence that suggests that both biological systems and social processes underlie gender patterns. [40,41] The programme also offered time to try out the new knowledge, to register it, and to review on experiences. GP registrars were offered time for new learning opportunities inside or outside general practice after a tutorial. We expected this teaching approach to be more fruitful to help GP registrars to realize and understand the importance of sex and gender aspects and differences in primary care.

The training programme as presented seems to work well but we have to point out some limitations. Our evaluation is not rigorous partly because of the low response rate. The assessment and ideas of the GP registrars who did not complete the survey, their assessment of the programme, and indeed their ideas about gender, may differ from those of the GP registrars who did complete the survey. Also, the evaluation did not include a sound assessment of the acquired knowledge and skills. This limits the scope of the evaluation of the programme. Demographic data of non-responders are not available, so comparisons between responders and nonresponders could not be performed. Furthermore, some of our GP registrars who were undergraduate students at Radboud University Nijmegen Medical Centre already have had the opportunity to learn about GSM by following courses in the undergraduate curriculum. Those GP registrars may therefore be somewhat more aware of gender-related issues than GP registrars who were undergraduate students at universities elsewhere.

The next step in incorporating GSM in medical education is to take new initiatives to teach also GP trainers and to include gender-related issues in GP registrars' examinations. GP trainers entrusted with the educational supervision of GP registrars have a special responsibility for inculcating the principles of good medical practice including gender-specific medicine. If the GP trainer does not share these new insights, it is hard to implement change for GP registrars. [42,43] Also, teaching and learning needs assessment. Current examinations in Dutch GP training do not systematically include gender-specific knowledge. Monitoring what has been learned is a good way of providing feedback. Teaching and learning GSM needs assessment of the applied knowledge of sex and gender differences in GP registrars' examinations.

## Conclusion

Teaching and learning gender-specific medicine is feasible in GP training. GP registrars found gender-specific medicine important and interested to learn. We recommend and encourage stakeholders of medical schools to provide learning opportunities in GSM to GP registrars.

## Competing interests

The authors declare no financial or non-financial competing interests other than they are members of department of primary care (Nijmegen) and social medicine (Maastricht) and wish to ensure a sustainable supply of general practitioners.

## Authors' contributions

TLJ developed the design of the study and the training programme. PD carried out the analysis and wrote the first draft of the manuscript. All authors were involved in critically revising the manuscript, and have read and approved the final manuscript.

## Pre-publication history

The pre-publication history for this paper can be accessed here:


